# Effectiveness of artemether–lumefantrine for treating uncomplicated malaria in low- and high-transmission areas of Ghana

**DOI:** 10.1186/s12936-024-04850-0

**Published:** 2024-02-05

**Authors:** Mawusi Adepa Mawuli, Linda Eva Amoah, Liwang Cui, Neils Ben Quashie, Yaw Asare Afrane

**Affiliations:** 1 West African Centre for Cell Biology of Infectious Pathogens (WACCBIP), College of Basic and Applied Sciences, University of Ghana, Legon, Accra, Ghana; 2 Department of Pathology, University of Ghana Medical School, College of Health Sciences, University of Ghana, Korle-Bu, Accra, Ghana; 3 Department of Immunology, Noguchi Memorial Institute for Medical Research College of Health Sciences, University of Ghana, Legon, Accra, Ghana; 4 Department of Internal Medicine, University of South Florida, 3720 Spectrum Blvd, Tampa, FL 33612 USA; 5 Department of Epidemiology, Noguchi Memorial Institute for Medical Research, College of Health Sciences, University of Ghana, Legon, Accra, Ghana; 6 Centre for Tropical Clinical Pharmacology and Therapeutics, University of Ghana Medical School, University of Ghana, Korle-Bu, Accra, Ghana; 7 Department of Medical Microbiology, University of Ghana Medical School College of Health Sciences, University of Ghana, Korle-Bu, Accra, Ghana

**Keywords:** Artemether–lumefantrine, Delayed parasite clearance Ghana, *Plasmodium falciparum*, Uncomplicated malaria

## Abstract

**Background:**

Artemisinin-based combination therapy (ACT) has been effective in the supervised treatment of uncomplicated malaria in Ghana. Since ACT usage is primarily unsupervised, this study aimed to determine the effectiveness of artemether–lumefantrine (AL) for treating malaria patients in two transmission settings in Ghana.

**Methods:**

Eighty-four individuals with uncomplicated *Plasmodium falciparum* malaria were recruited from Lekma Hospital (LH) in Accra (low-transmission area; N = 28), southern Ghana, and King’s Medical Centre (KMC) in Kumbungu (high-transmission area; N = 56), northern Ghana. Participants were followed up for 28 days after unsupervised treatment with AL. The presence of asexual parasites was determined by microscopic examination of Giemsa-stained blood smears. *Plasmodium* species identification was confirmed using species-specific primers targeting the *18S rRNA* gene*.* Parasite recrudescence or reinfection was determined by genotyping the *Pfmsp* 1 and *Pfmsp* 2 genes.

**Results:**

After AL treatment, 3.6% (2/56) of the patients from KMC were parasitaemic on day 3 compared to none from the LH patients. One patient from KMC with delayed parasite clearance on day 3 remained parasite-positive by microscopy on day 7 but was parasite-free by day 14. While none of the patients from LH experienced parasite recurrence during the 28-day follow-up, three and two patients from KMC had recurrent parasitaemia on days 21 and 28, respectively. Percentage reduction in parasite densities from day 1, 2, and 3 for participants from the KMC was 63.2%, 89.5%, and 84.5%. Parasite densities for participants from the LH reduced from 98.2%, 99.8% on day 1, and 2 to 100% on day 3. The 28-day cumulative incidence rate of treatment failure for KMC was 12.8% (95% confidence interval: 1.9–23.7%), while the per-protocol effectiveness of AL in KMC was 89.47%. All recurrent cases were assigned to recrudescence after parasite genotyping by *Pfmsp* 1 and *Pfmsp* 2.

**Conclusion:**

While AL is efficacious in treating uncomplicated malaria in Ghana, when taken under unsupervised conditions, it showed an 89.4% PCR-corrected cure rate in northern Ghana, which is slightly below the WHO-defined threshold.

**Supplementary Information:**

The online version contains supplementary material available at 10.1186/s12936-024-04850-0.

## Background

Over the past decade, significant progress has been made toward malaria control. Despite this, malaria continues to be a global health burden, with an estimated 241 million cases and 627 thousand deaths in 2020 [1]. Sub-Saharan Africa accounted for approximately 96% of the total deaths due to malaria. In Ghana, a 2021 report from the National Malaria Control Programme indicated that for every one thousand hospitalizations, 161 were due to malaria infection, and malaria accounted for 68 of one thousand deaths. *Plasmodium falciparum* accounts for 90–98% of malaria infections [2–4].

Artemisinin-based combination therapy (ACT) is recommended for the treatment of uncomplicated falciparum malaria. Children and adults, except pregnant women in their first trimer, can be prescribed any of the artemisinin-based combinations: artemether + lumefantrine (AL), artesunate + amodiaquine (AS–AQ), artesunate + mefloquine (AS–MQ), dihydroartemisinin + piperaquine (DHA–PQ), and artesunate + sulfadoxine–pyrimethamine (AS–SP).

Ghana adopted ACT as the frontline treatment of uncomplicated *P. falciparum* malaria in 2004 [5]. Currently, AS–AQ and AL are the recommended first-line ACT, and DHA–PQ is the alternative ACT [6]. Adverse effects such as vomiting, is frequently reported with use of AS–AQ as compared to AL, leading to AL being commonly used in Ghana [7, 8]. Artemisinin and its derivatives are essential components of antimalarial treatment because they kill both young and mature intraerythrocytic parasites [9, 10]. In addition, artemisinins are known to have some gametocidal activities [11]. However, some studies reported the presence of gametocytes after treatment with ACT, indicating that the administration of artemisinin-based combinations may impact malaria transmission [12].

The World Health Organization (WHO) recommends a cure rate of > 90% for ACT [9]. However, a much lower cure rate of 77.8% has been reported in Burkina Faso after unsupervised ACT administration [13]. Some studies have also reported the presence of high/low residual sub microscopic parasitaemia on day 3 after supervised artemisinin-based combination treatment [12, 14]. Residual sub-microscopic infections after treatment with artemisinin-based combinations may cause recrudescent infections [12]. In vivo artemisinin resistance is determined by parasite clearance time (PCT) and its related clinical phenotype, delayed parasite clearance [15]. However, accurate determination of parasite clearance half-life requires 6- or 8-hourly sampling of parasitaemia [16], which is requires hospitalization of participants, or participants return to study site for follow up. In practice this is a challenge as it requires more resources or logistics. As a result, the presence of malaria parasites in a 72-h blood smear after the initiation of treatment, which requires a single time-point measurement and fewer resources, has been proposed as a simple predictor of artemisinin resistance [17].

Artemisinin-based combinations administered under the strict supervision of drug intake have been efficacious in Ghana, with overall cure rates of 99.2% and 96% for ASAQ and AL, respectively [18]. Although case management of malaria in Ghana recommends completion of the ACT course, artemisinin-based combinations and other anti-malarials are available as over-the-counter drugs in local pharmacies as well as in health facilities, which are prescribed mostly as unsupervised treatment. Unsupervised treatment is expected to result in variations in drug efficacy as a result of, for example, nonadherence to dosage and time of treatment and noncompletion of treatment. Thus, the treatment outcome of malaria patients receiving unsupervised ACT may reflect the true drug efficacy in clinical practice. Therefore, the current study aimed to assess the effectiveness of AL for the unsupervised treatment of malaria in low- and high-transmission settings in Ghana.

## Methods

### Study sites

**Fig. 1:**
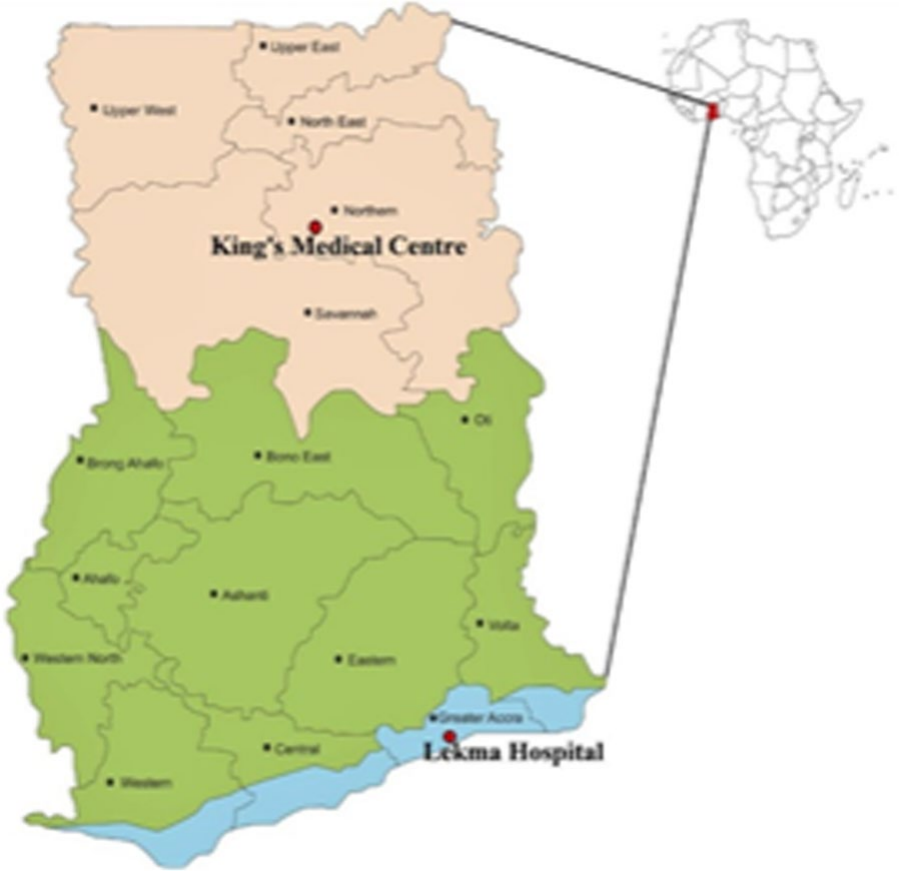
Map of Ghana showing the locations of the two study sites, King’s Medical Centre (KMC) and Lekma Hospital (LH). (Note: this is an author-edited version of the map of Ghana)

Patients were recruited from King’s Medical Centre (KMC) in Kumbungu District (9° 34′ 36.3″ N 0° 59′ 39.2″ W), a high-transmission area in rural northern Ghana, and Lekma Hospital (LH) in Ledzokuku-Krowor Municipality (5° 36′ 04.0″ N 0° 11′ 09.1″ W), an urban low-transmission area within the city of Accra in southern Ghana (Fig. 1). Prevalence of malaria in northern Ghana and Accra have been reported to be 13.0% and 2.4%, respectively [19]. Kumbungu District has a unimodal rainfall pattern that starts from May to the end of November, with the peak occurring from July to September. The average rainfall is approximately 1000 mm. The Ledzokuku-Krowor area has two peak rainy seasons; the long rainy season is from April to June, and the second is from September to October. The average rainfall and temperature are 730 mm and 26.8 °C, respectively [20].

### Sample size estimation

The prevalence of clinical malaria in Ghanaian adults and children has been reported to be 14% and 12%, respectively [21, 22]. Reference to the formula for calculating sample size in prevalence studies (Daniel, 1999) n = Z^2^P (1 − P)/d^2^, where n = sample size; Z (1.96) is the statistic corresponding to the level of confidence, P is the expected prevalence and d is the precision. Using a prevalence of clinical malaria of 1 4% and precision of 0.09, the estimated sample size was 57.

### Recruitment of participants

The study was performed between August and November 2017 at both study sites. During this time, individuals between the ages of 1 and 35 years reporting to the above-mentioned health facilities with symptoms of uncomplicated malaria were recruited. Individuals with uncomplicated malaria were defined as having an axillary temperature ≥ 37.5 °C or a history of fever within the last 24 h, a positive test for malaria parasites by microscopy at any parasitaemia, but lacking symptoms of severe malaria. A haemoglobin concentration of > 5 g/dL of blood was also considered for inclusion. Individuals who had taken any antimalarial drugs 2 weeks before the study were also excluded.

### Malaria diagnosis and parasite counts by microscopy

Thick and thin blood smears were stained with 4% buffered Giemsa solution (pH 7.1) for 30 min. Parasite density per microlitre of blood was determined by counting the asexual malaria parasites per 200 white blood cells (WBCs) on the thick film under oil emersion using a light microscope and assuming a white cell count of 8000/μL. Gametocyte densities were also counted on the thick film per 500 WBCs. A smear was reported as negative if no malaria parasites were observed after viewing 100 high-power fields. Two qualified microscopists read the smears, and if there were discrepancies (> 50% difference in parasite counts) in parasite counts, a third microscopist was consulted. The average of the two closest counts was taken as the parasite count for that case.

### Treatment and follow-up

Participants were prescribed AL (Coartem® Novartis; 20 mg/120 mg per kg body weight) in a 6-dose regimen by clinicians per body weight as follows: 1 tablet per treatment for weights 5 to < 15 kg; 2 tablets per treatment for 15–20 kg; 3 tablets per treatment for weights between 25 and 35 kg, and 4 tablets per treatment for weights > 35 kg. There was no weight cut-off. Participants were advised to strictly take the medication at 0, 8, 24, 36, 48, and 60 h with food. All treatments occurred at home without supervision.

Patient follow-up was performed on days 1, 2, 3, 7, 14, 21, and 28. Blood samples (~ 50 µL) were collected from the study participants by a finger prick during admission (day 0) and on the days of the follow-up and used to prepare thick and thin blood smears and filter paper blood blots. Blood blots were air-dried and kept in tightly sealed Ziploc bags containing silica and stored at room temperature until use. There was no randomization or blinding of participants for the treatment. Participants who were parasitaemic after day 3 did not receive any treatment.

### Study outcomes

Treatment outcomes were defined according to the 2009 WHO methods for survival of anti-malarial drug efficacy [23]. Study participants who were not parasitaemic on day 28, had no fever and were not previously classified as having early treatment failure, late clinical failure or late parasitological cure were described as individuals with adequate clinical and parasitological responses. Participants were described as having early treatment failure when they were parasitaemic on day 3 and had fever. Late treatment failure was used to describe participants who were parasitaemic on day 28 with or without fever and were not previously classified as having early treatment failure, late clinical failure or late parasitological cure.

### Primary study outcome

The primary efficacy indicator for this study involved the identification of participants with recrudescence infections per total number of participants who completed the study. All cases that were lost to follow-up, had new infections and results that were indeterminate were excluded from the analyses.

### Parasite detection and species identification by PCR

The saponin-Chelex method was used with slight modifications to extract DNA from dried blood blots [24]. Briefly, cells were lysed with 0.05% saponin in phosphate-buffered saline (PBS) at 4 °C overnight, followed by incubation in 10% Chelex with intermittent washing steps with distilled water. DNA was eluted with 50 µL of DNase/RNase-free water and kept at − 20 °C until use. *Plasmodium* species were determined by nested PCR using genus- and species-specific primers that target the *18S rRNA* gene [25]. All reactions were carried out in a volume of 15 μL with 5 μL of DNA template for the first reaction and 0.5 μL of the first reaction product for the nested reaction. The master mix contained 167 nM dNTPs, 2.5 nM MgCl_2_, 80 nM of each primer, and 1 U of One Taq polymerase (New England BioLabs Inc.). The PCR conditions were as follows: initial denaturation for 5 min at 94 °C, followed by 35 cycles of 30 s at 94 °C, 1 min at 55 °C (58 °C for the nested PCR), and 1 min at 68 °C, and a final extension for 5 min at 68 °C. The 3D7 *P. falciparum* strain was used as a positive control, and no template was used as a negative control.

### Genotyping of *Plasmodium* parasites to distinguish between recrudescence and new infections

*Plasmodium falciparum* was genotyped by amplifying the polymorphic regions of block 2 of the *merozoite surface protein 1* (*msp1*) gene and block 3 of the *msp2* gene [26]. Nested PCR with allele-specific primers [27] was used to distinguish three major allelic families (K1, MAD20, and RO33) for *msp1* and two major allelic families (FC27 and IC1/3D7) for *msp2*. Positive control samples were prepared from cultured laboratory clones of 3D7, RO33 and FC27 (Additional file 1). Variations in the length of the amplified fragments were identified following agarose gel electrophoresis (Additional file 1). MSP 1 and 2 genotyping were performed for individuals with recurrent parasitaemia on or after day 7. All paired samples (day 0 and day of parasite recurrence) were run side by side on the same gel. If all alleles (in at least 1 locus) in parasites from the posttreatment sample were different from those in the day 0 sample, parasites in the posttreatment sample were classified as a new infection. A difference in base pairs of at least 10 was considered to indicate 2 different alleles [28]. If at least one allele at each locus was common to the paired samples, the posttreatment sample was classified as a recrudescent sample.

### Statistical analysis

Data were entered and analysed in SPSS version 20. The Z test was used to compare the differences in proportions between participants from the high- and low-transmission areas. An independent *t* test was used to compare the difference in age of participants and parasite densities. Pearson’s correlation was used to test for correlation between categorical data (e.g., the difference in the mean age of participants who carried gametocytes). A p value of less than 0.05 was regarded as statistically significant.

## Results

### Patients’ demographic and clinical profiles

A total of 84 malaria patients, including 56 from the high-transmission area (KMC) and 28 from the low-transmission area (LH), were recruited into the study. The patients from KMC were significantly older than those from LH (Table 1). Participants from the KMC presented with significantly higher axillary temperatures than those from the LH. The mean asexual parasite density for participants from KMC was lower than that for participants from LH, although the difference was not statistically significant. While four (7.1%) patients from KMC carried gametocytes at recruitment, none from LH were gametocyte-positive by microscopy on day 0 (Table 1). A comparison of the ages of participants who carried asexual parasites and those who carried gametocytes showed that younger children (mean ± standard deviation; 2.0 ± 0.7) were more likely to carry gametocytes than older children (8.5 ± 1.1) (correlation test − 0.74; p = 0.03).

**Table 1:** Demographic and clinical characteristics of study participants from the two study sites

	KMC (N = 56)	LH (N = 28)	p value
Gender (% male)	46	50	< 0.0001
Mean age in years (range)	9.4 (1–35)	5.39 (2–17)	0.0343
Mean axillary temperature (°C) (range)	37.7 (36.7–39.6)	38.4 (35.8–40.4)	< 0.0001
Mean asexual parasite density (parasites/µL of blood) (range)	48,615.86 (480–282,000)	71,869 (6000–490,000)	< 0.0001
Mean sexual parasite density (gametocytes/µL of blood) (range)	2.57 (16–48)	–	–

P values were calculated using differences in mean values or differences in proportions with Medcalc

### Primary outcomes

**Fig. 2:**
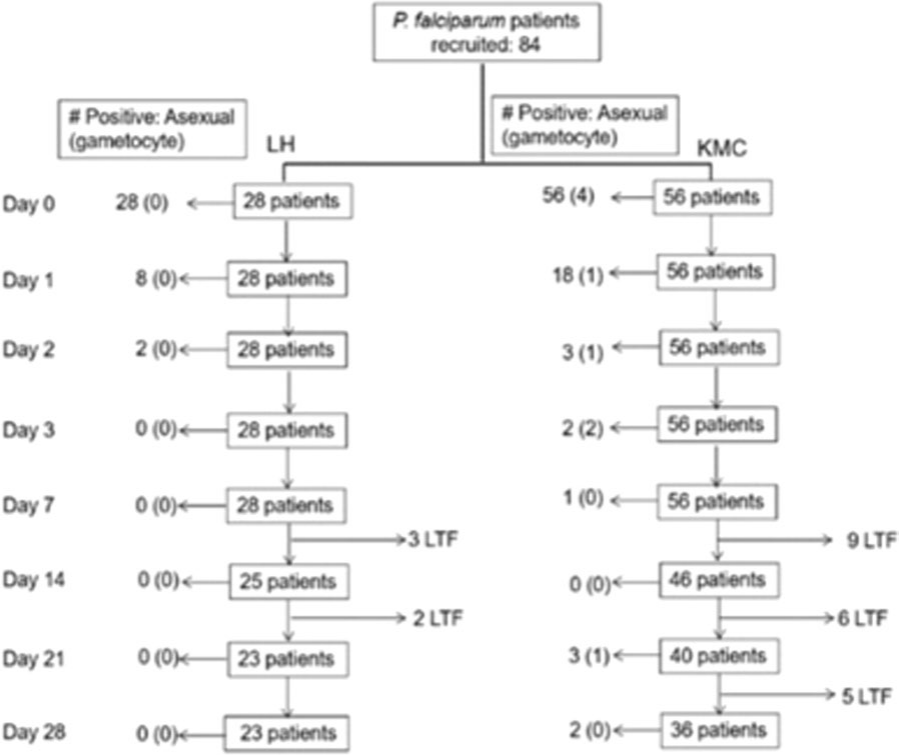
Flow chart of treatment outcome with artemether–lumefantrine of patients with uncomplicated malaria from KMC and LH, Ghana. *LTF* loss to follow-up

Out of the total number (84:56 from KMC and 28 from LH) of participants recruited, 59 (70.2%) participants; 36 (64.29%) from KMC and 23 (82.1%) from LH) were successfully (followed for 28 days (Fig. 2).

**Fig. 3:**
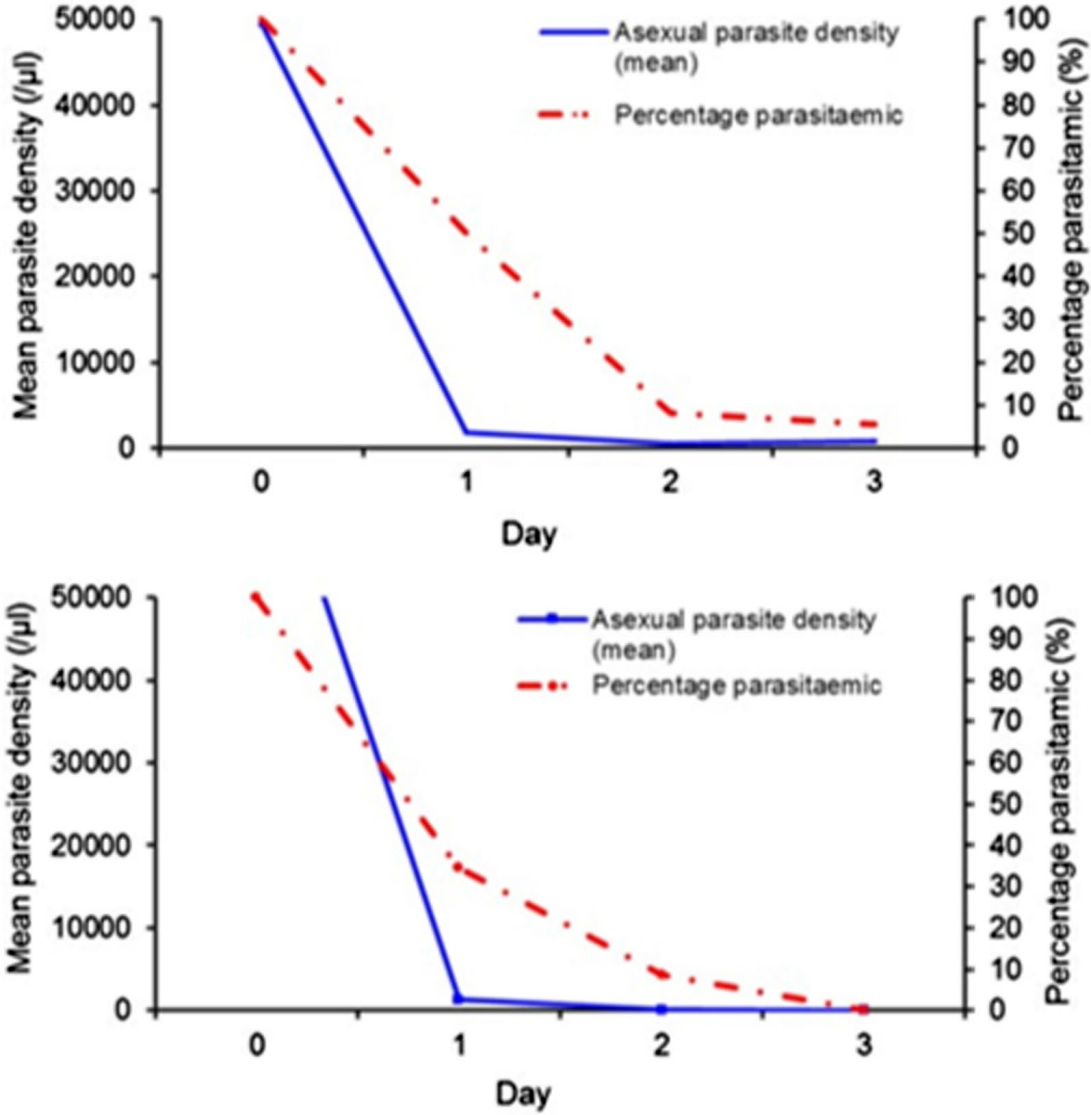
Mean asexual parasite density (blue unbroken line) and percentage parasitaemic (%) (red broken line) participants in KMC (**A**) and LH (**B**)

All patients from LH cleared parasitaemia by day 2. In comparison, two out of 56 (3.6%) patients from KMC were parasitaemic on day 3 (Fig. 3). None of the participants who were parasitaemic on day 3 had fever. One case from KMC was considered late treatment failure: the patient was parasitaemic on day 3, remained parasitaemic on day 7 but was aparasitaemic on day 14. Whereas recurrent parasitaemia was not observed in LH during the 28-day follow-up, it was identified in 4 of the 56 KMC patients. These four recurrent cases from KMC were late parasitological failures, with three occurring on day 21, one of which remained parasitaemic on day 28 and 1 of which developed parasites on day 28. This resulted in a cumulative incidence rate of recurrence of 12.8% (95% confidence interval: 1.9–23.7%) (Fig. 3).

**Fig. 4:**
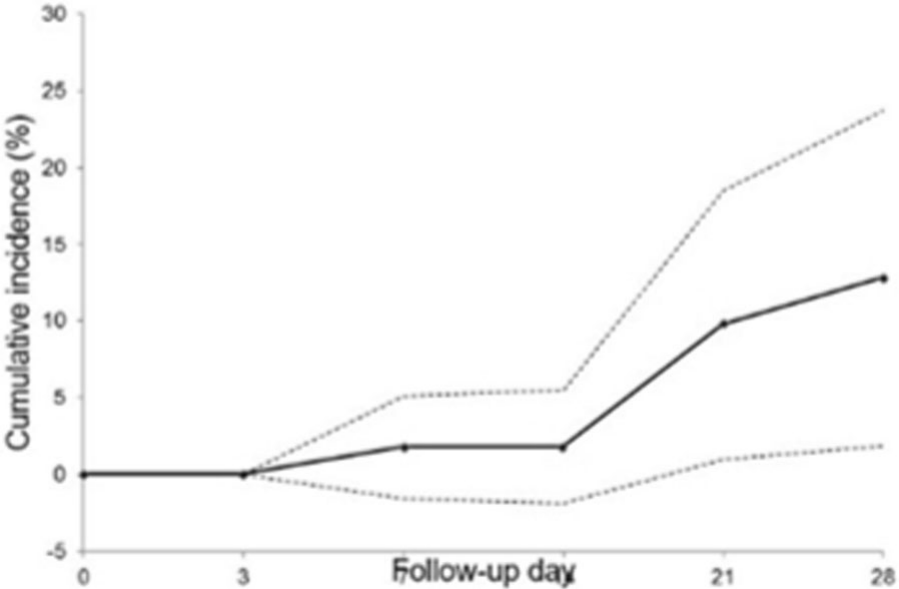
Cumulative incidence of recurrent patients in 28 days in KMC. Dashed lines indicate the 95% confidence interval

Overall, 34 participants from KMC and all participants from LH had adequate clinical and pathological responses (Fig. 4). The cure rate (day 28) of AL at KMC was 89.5% (34/38) compared to 100% at LH. All recurrent cases identified on days 7, 21, and 28 were classified as recrudescence after genotyping *pfmsp1* and *msp2.* Thus, the PCR-corrected cure rate of AL at KMC remained at 89.4% **(**95% CI—the confidence interval for PCR-corrected efficacy is 23.546 to 47.512).

## Discussion

The efficacies of artemisinin-based combinations against uncomplicated *P. falciparum* malaria have been monitored at multiple sentinel sites across Ghana [18, 29, 30]. While these studies were carried out under more stringent clinical monitoring, the effectiveness of AL in practice was rarely evaluated. Thus, this study aimed to determine the effectiveness of unsupervised AL treatment in low and high transmission areas in Ghana. The results showed that AL remained highly effective at the southern site (LH) with a 100% cure rate, whereas it was suboptimal at the northern site, with a PCR-corrected cure rate of 89.4%. Such regional differences have also been observed during the monitoring of AL efficacies. While the overall efficacy of AL was above 95% in Ghana, two coastal regions have experienced a decline in efficacy in recent years, with PCR-corrected cure rates of AL approaching the 90% threshold set by WHO [18]. With the substantial heterogeneity of malaria epidemiology across Ghana, it is important to monitor ACT efficacies/effectiveness in multiple sentinel sites.

Day 3 parasite positivity is used as a proxy for artemisinin resistance, with 10% and 5% set as the thresholds for Southeast Asia and Africa, respectively [31]. In KMC, 3.6% of patients remained parasitaemic on day 3. However, it is noteworthy that the 28-day PCR-corrected cure rate declined to 86.1%, and one of the cases was an early parasitological failure. An earlier ACT clinical efficacy study conducted in the same region detected a 90.4% PCR-corrected cure rate for AL [29]. Compared to the directly observed treatment performed in the previous study, the current study with unsupervised AL treatment may have compliance issues, undermining the study results. However, the daily blood smear monitoring activities of the research team during the 3-day ACT unsupervised administration should have a positive effect on compliance. In addition, some of the recurrent cases had gametocytaemia, highlighting the potential for subsequent transmission. Altogether, these studies emphasize the significance of continuous monitoring of ACT, taking into account the transmission intensity in an area. Results from this study shows that AL continuous to be effective in the low transmission area, whereas there was the occurrence of treatment failures or participants from the high transmission area.

Clinical manifestations of malaria are significantly influenced by transmission intensity, age and acquired immunity to the parasite [32, 33]. In low transmission settings, exposure to malaria parasites is less as compared to high transmission settings. The transmission intensity would, therefore, influence the exposure to parasite hence affect the acquired immunity to malaria [34]. This explains the occurrence of high parasitaemia and high axillary temperature in a relatively younger age for participants from LH as compared to KMC. Previous studies in Ghana observed a decline in the severity of clinical manifestations of malaria with increasing transmission intensity [35]. In light of this, when instituting malaria control measures, transmission intensity must be considered. In low endemic settings, this could further reduce parasite circulating in the area and would negatively impact the acquisition of natural immunity to malaria.

## Conclusion

AL, as an unsupervised treatment, remained effective against uncomplicated falciparum malaria in a southern region, whereas declined effectiveness was observed in a northern region. Given the possible impact of compliance issues for unsupervised treatment, it is encouraged that healthcare practitioners, particularly pharmacists and dispensers of anti-malarial drugs, explain the significance of completing the dosage at appropriate times for the intake of medications. This study also underlines the need for continuous monitoring of the clinical efficacy/effectiveness of ACT in multiple sentinel sites in Ghana.

### Supplementary Information

**Additional file 1.** Gel images of MSP1 and MSP 2 genotyping.

## Data Availability

Data supporting the conclusions of this article are included within the article. The datasets analysed are available upon request to the corresponding author.

## Abbreviations

ACT: Artemisinin-based combination therapy
CQ: Chloroquine
SP: Sulfadoxine–pyrimethamine
PCT: Parasite clearance time
AS-AQ: Artesunate–amodiaquine
AL: Artemether–lumefantrine
DH-PQ: Dihydroartemisinin–piperaquine
WHO: World Health Organization
NMIMR: Noguchi Memorial Institute for Medical Research
GHS: Ghana Health Service
KMC: King’s Medical Centre
LH: Lekma Hospital
DNA: Deoxyribonucleic acid
MSP: Merozoite surface protein
