# Supplementary material for: Effectiveness of artemether–lumefantrine for treating uncomplicated malaria in low- and high-transmission areas of Ghana
Source: Malar J. 2024 Feb 5;23:40. doi: 10.1186/s12936-024-04850-0 (PMC10845584; doi:10.1186/s12936-024-04850-0)
Supplement: Supplementary file 1 — Additional file 1. Gel images of MSP1 and MSP 2 genotyping. [file 12936_2024_4850_MOESM1_ESM.ppt]

## Slide 1
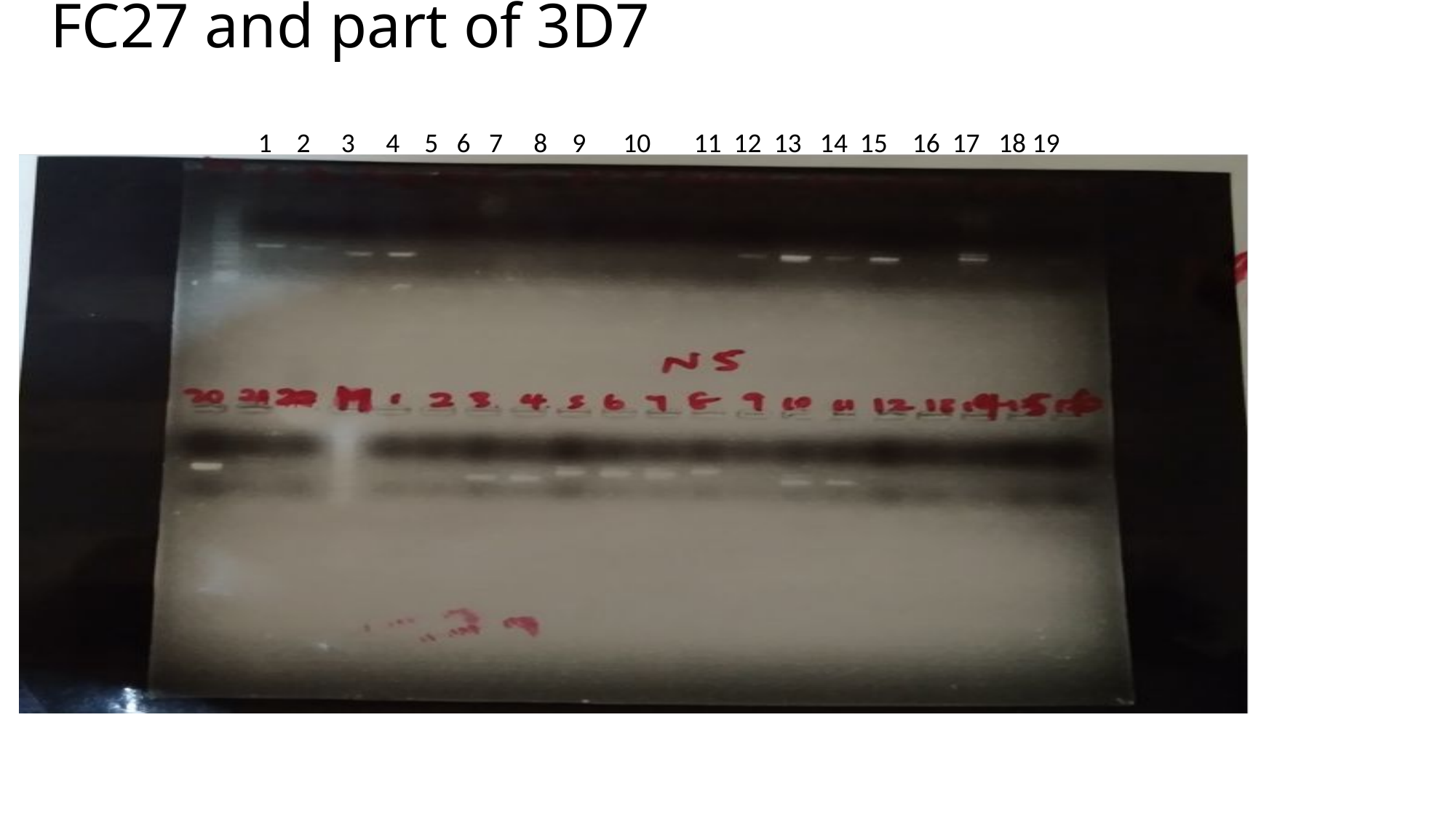

# FC27 and part of 3D7
1 2 3 4 5 6 7 8 9 10 11 12 13 14 15 16 17 18 19

## Slide 2
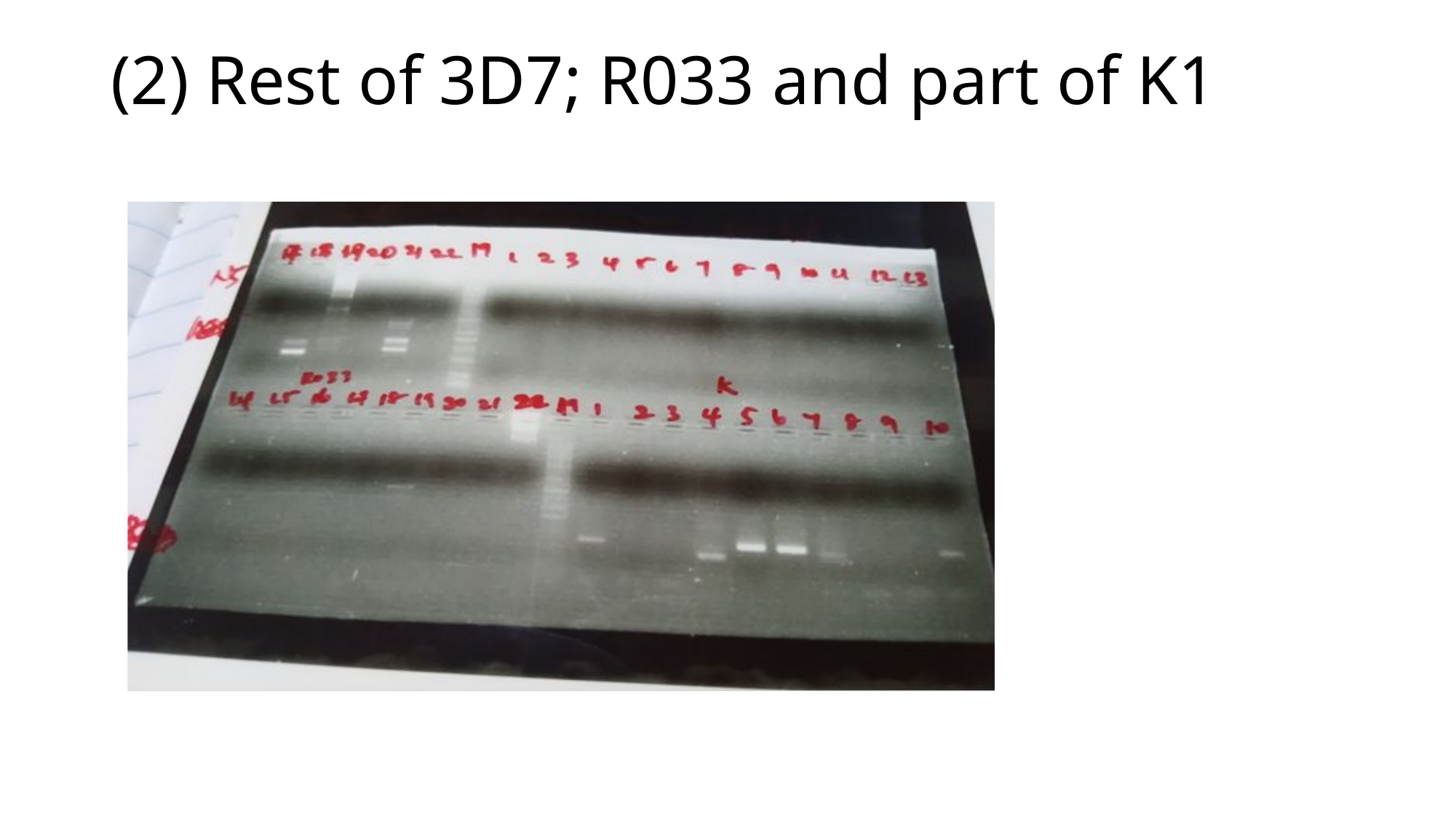

# (2) Rest of 3D7; R033 and part of K1

## Slide 3
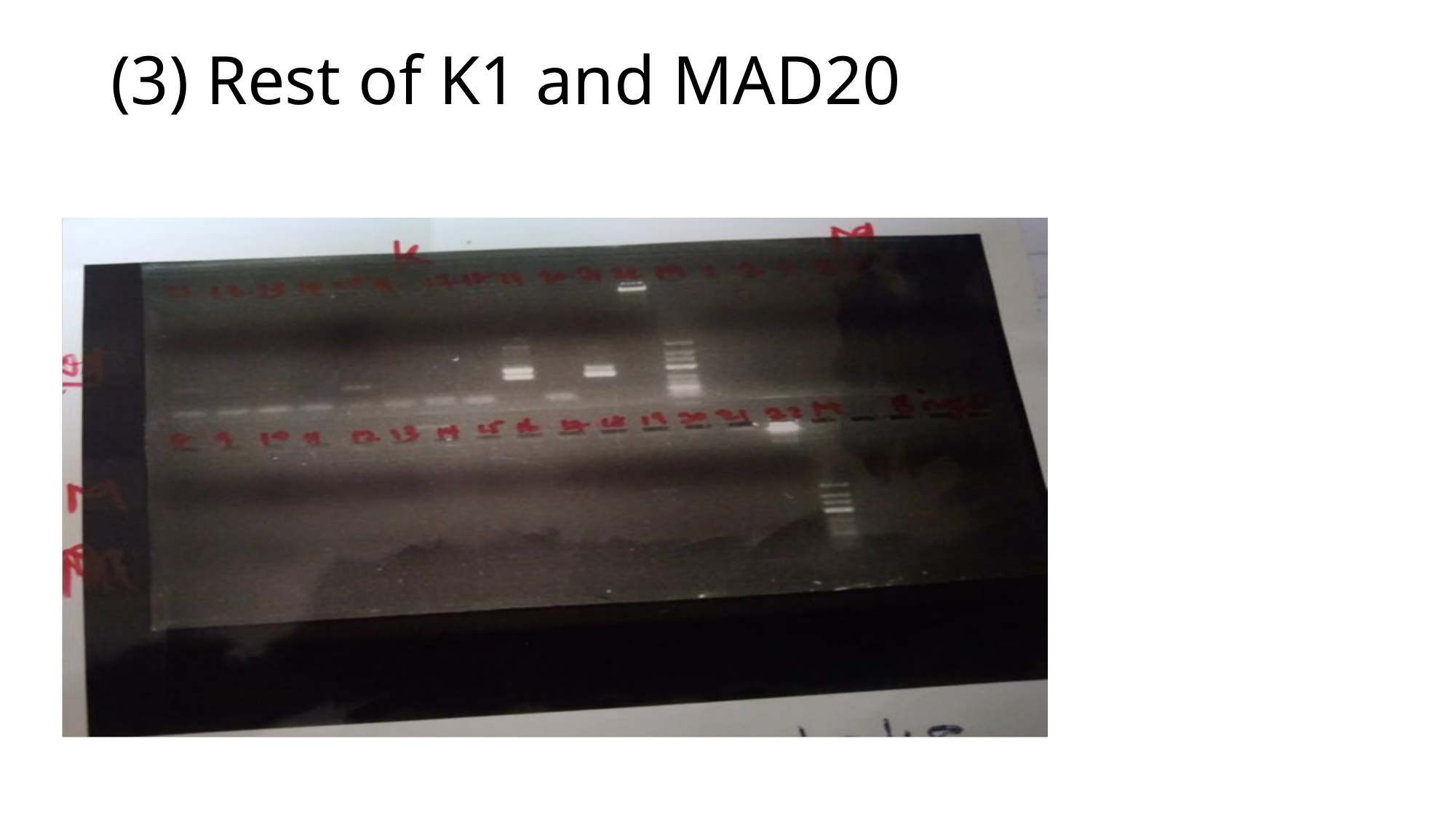

# (3) Rest of K1 and MAD20

## Slide 4
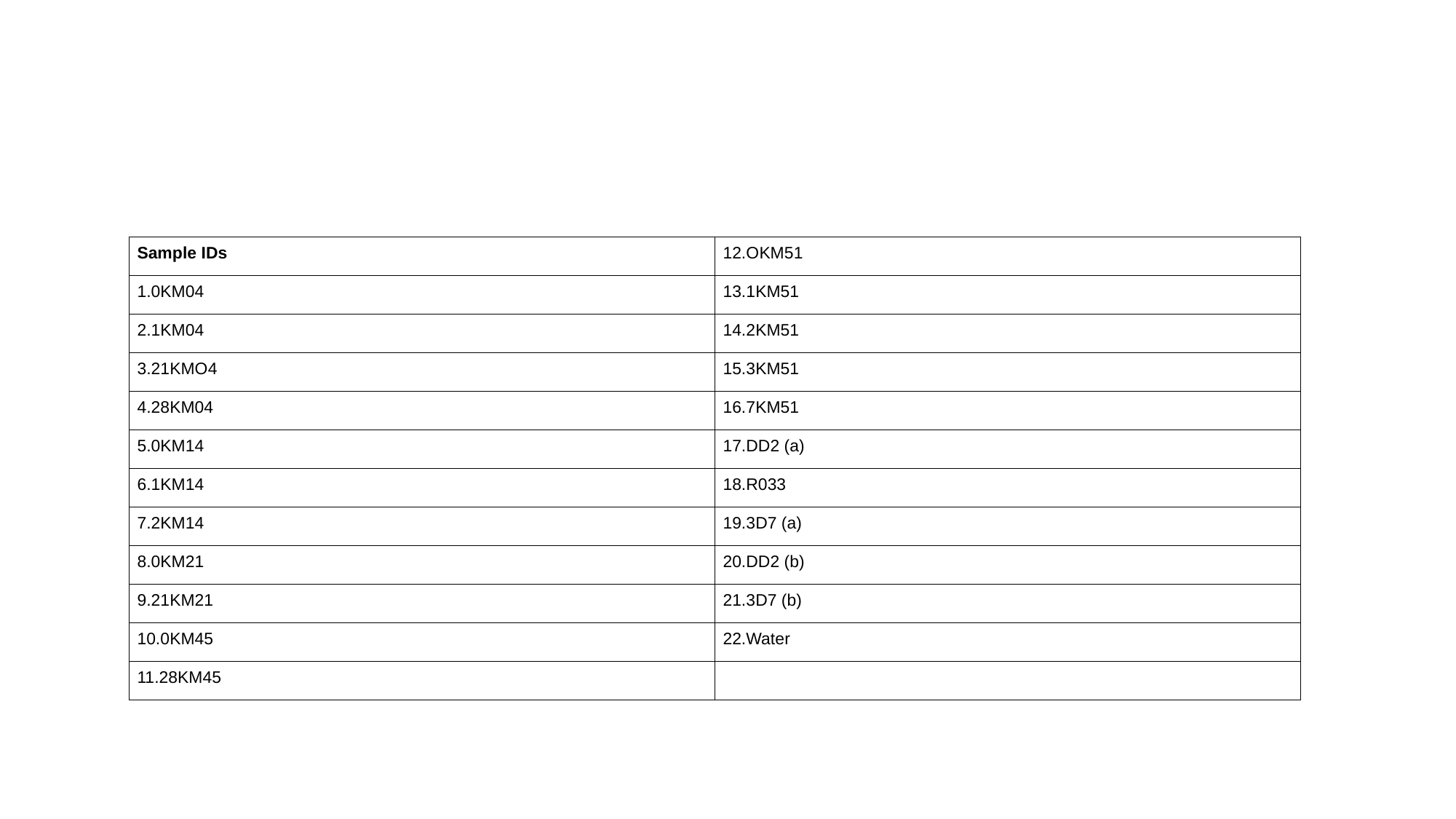

#
| Sample IDs | 12.OKM51 |
| --- | --- |
| 1.0KM04 | 13.1KM51 |
| 2.1KM04 | 14.2KM51 |
| 3.21KMO4 | 15.3KM51 |
| 4.28KM04 | 16.7KM51 |
| 5.0KM14 | 17.DD2 (a) |
| 6.1KM14 | 18.R033 |
| 7.2KM14 | 19.3D7 (a) |
| 8.0KM21 | 20.DD2 (b) |
| 9.21KM21 | 21.3D7 (b) |
| 10.0KM45 | 22.Water |
| 11.28KM45 | |

## Slide 5
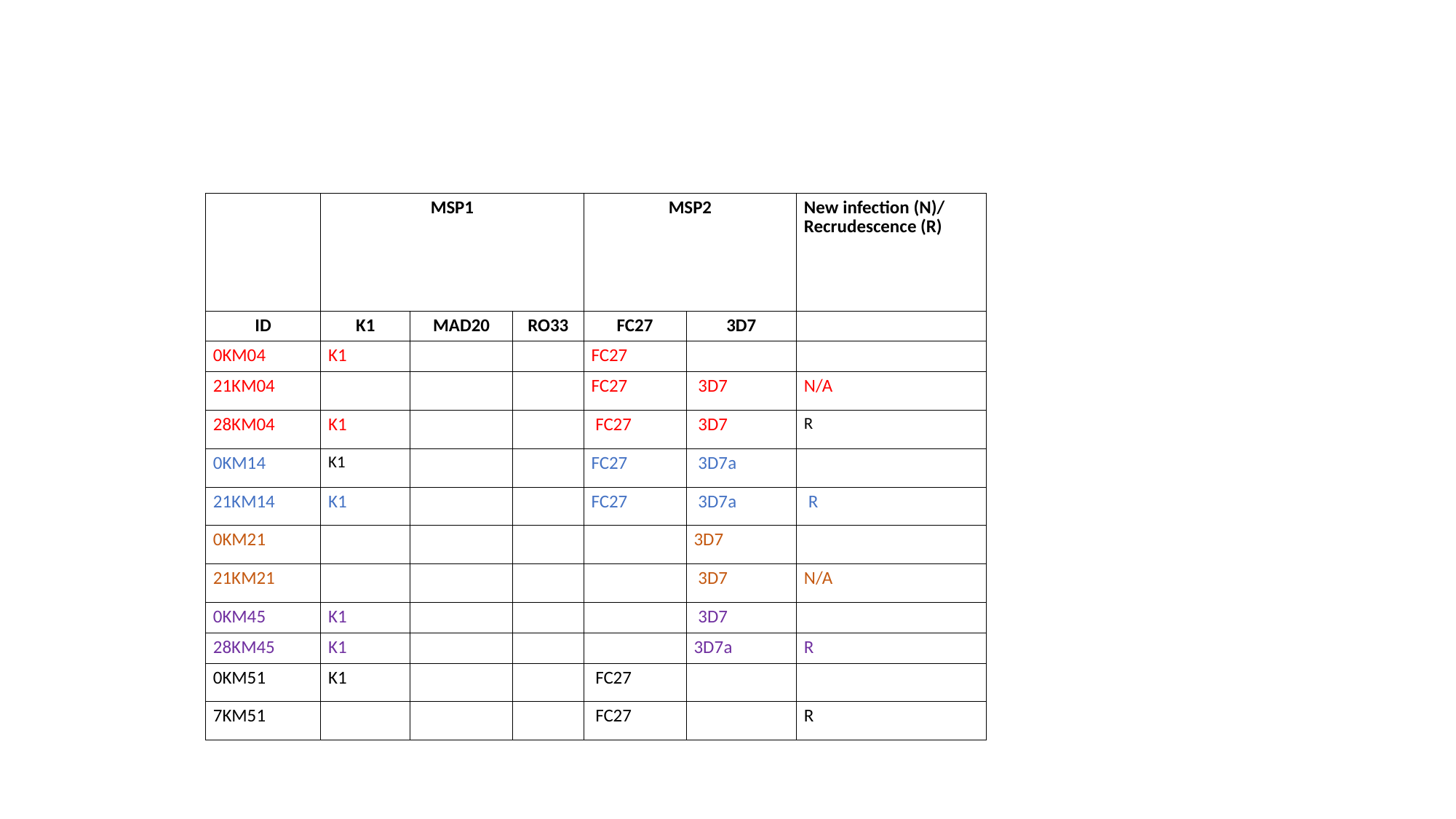

| | MSP1 | | | MSP2 | | New infection (N)/ Recrudescence (R) |
| --- | --- | --- | --- | --- | --- | --- |
| ID | K1 | MAD20 | RO33 | FC27 | 3D7 | |
| 0KM04 | K1 | | | FC27 | | |
| 21KM04 | | | | FC27 | 3D7 | N/A |
| 28KM04 | K1 | | | FC27 | 3D7 | R |
| 0KM14 | K1 | | | FC27 | 3D7a | |
| 21KM14 | K1 | | | FC27 | 3D7a | R |
| 0KM21 | | | | | 3D7 | |
| 21KM21 | | | | | 3D7 | N/A |
| 0KM45 | K1 | | | | 3D7 | |
| 28KM45 | K1 | | | | 3D7a | R |
| 0KM51 | K1 | | | FC27 | | |
| 7KM51 | | | | FC27 | | R |
